# Alcohol diagnostic validation for injury-related trauma: Findings from a pilot study

**DOI:** 10.1177/20552076231218138

**Published:** 2023-12-03

**Authors:** Megan Prinsloo, Petal Petersen Williams, Ian Neethling, Shibe X Mhlongo, Sithombo Maqungo, Margaret M Peden, Charles Parry, Richard Matzopoulos

**Affiliations:** 1 Burden of Disease Research Unit, 59097 South African Medical Research Council, Cape Town, South Africa; 2 Institute for Lifecourse Development, Faculty of Education, Health & Human Sciences, University of Greenwich, UK; 3 School of Public Health, University of Cape Town, Cape Town, South Africa; 4 Mental Health, Alcohol, Substance Use and Tobacco Research Unit, 59097 South African Medical Research Council, Cape Town, South Africa; 5 Department of Psychiatry and Mental Health, University of Cape Town, South Africa; 6 Institute for Life Course Health Research, Department of Global Health, Stellenbosch University, South Africa; 7 Gender and Health Research Unit, 59097 South African Medical Research Council, Cape Town, South Africa; 8 Orthopaedic Trauma Service, Groote Schuur Hospital, University of Cape Town, South Africa; 9 George Institute for Global Health, Imperial College London, UK; 10 WHO Collaborating Centre on Injury Prevention and Trauma Care, Geneva, Switzerland; 11 Department of Psychiatry, Stellenbosch University, Cape Town, South Africa

**Keywords:** alcohol consumption, alcohol assessment tools, blood alcohol, evidentiary breath alcohol testing, breathalyzer, violence, injury, South Africa

## Abstract

**Introduction:**

Alcohol consumption is a key driver of the burden of violence and injury in South Africa (SA). Hence, we aim to validate various alcohol assessment tools against a blood test to assess their utility for improving national health practice and policy.

**Methods:**

We conducted a cross-sectional pilot study from 3 to 19 August 2022 at Groote Schuur Hospital in Cape Town, SA. This was to test logistics for the time of venous blood centrifugation and validation of alcohol assessment tools used in injured patients ahead of the main validation study. Adults aged 18 years and older, who were injured <8 h before arrival were included. Consent was obtained for venous blood alcohol testing to validate, as the gold standard, against the following: active- and passive breath alcohol testing, clinical screening and a finger prick test. Descriptive statistics were reported for the pilot study.

**Results:**

The active breath alcohol test's digital reading and the passive test's ‘yes/no’ results corresponded well against the venous blood alcohol results. The average time to centrifugation was within the laboratory's 2-h cut-off requirement to preserve the alcohol in the serum.

**Discussion and Conclusion:**

The pilot study was helpful in identifying challenges with one of the alcohol assessment tools and prevented further costs ahead of the main validation study. We also determined that the selected tertiary hospital site caused a delay in recruiting eligible patients due to other hospital referrals. Hence, the main validation study is in progress at a district-level hospital for a larger sample of eligible patients for testing.

## Introduction

In high-violence settings such as South Africa (SA), there is a need for rapid and affordable diagnostic assessment tests to monitor alcohol consumption, a key driver of the burden of violence and injury, which impacts health system resources. The nature and extent of injury mortality in SA is well-documented, with extremely high rates of violence and road traffic deaths.^1,2^ Unfortunately, systems for capturing non-fatal injury and especially the impact of alcohol consumption preceding such incidents in SA are lacking.

Globally, reasons for such challenges include the difficulty in assessing blood alcohol concentration (BAC) because of the time-lapse after the incident^3,4^ and the lack of appropriate alcohol assessment tools in the emergency settings to accurately screen patients for their use of alcohol.^4^ The merits and challenges for particular alcohol assessment tools are well-documented. Venous blood for the detection of ethanol by gas chromatography (GC) is considered the ‘gold standard’^5^ for its ability to separate ethanol from other alcohol, but it is very costly. A clinical assessment utilizing ICD-10 Y91 coding for motor coordination, speech impairment and horizontal gaze nystagmus, among others, observes apparent patient intoxication.^6^ This was shown to perform well against breath alcohol concentrations (BrACs) using a breathalyzer in the emergency room (ER) setting but with lower concordance in patients who had consumed alcohol within six hours before injury. The experience and ability of the clinical observer to observe an individual's behaviour, as well as possible drug use in some injured patients, were thought to have influenced the accuracy of such observations.^7^ Breathalyzers are deemed less invasive and more cost-effective in measuring the presence of breath alcohol but have not always performed well compared to BAC testing.^8^ This could be due to patients being too intoxicated to provide deep breaths for blowing into the mouthpiece of such tools or too severely injured to co-operate, which are aspects to explore further. Personal breathalyzers linked to Bluetooth technology were less accurate than police-grade breathalyzers used during road-side testing.^9^

A global study identified the disproportionate burden attributable to alcohol consumption in low-to-middle income countries (LMICs).^10^ Yet, a systematic review of alcohol control interventions could not identify any studies from LMICs.^11^ Given the high burden of trauma in SA on the health system, the utility of the routine use of cost-effective tools to measure the burden of alcohol – for policy advocacy, should be assessed. This could aid in monitoring the impact of alcohol policy reforms more broadly.

Hence, we are currently conducting a study which aims to validate various alcohol assessment tools against a venous laboratory blood test by testing injured patients’ alcohol use to assess the utility of such devices for improving national health practice and policy. This paper describes the pilot study's findings, which was conducted to test courier logistics for venous blood testing and ease of use for alcohol assessment tools in a trauma setting, ahead of the current validation study.

## Methods

### Study design

We conducted a cross-sectional pilot study at Groote Schuur Hospital (a tertiary hospital in Cape Town, South Africa) from 3 to 19 August 2022.

### Population and sampling

Adult patients, 18 years and older, who presented with first-time treatment for an injury that occurred <8 h before arrival (to detect the presence of blood alcohol) were eligible for inclusion. Cognitively impaired adult patients were excluded. While the main validation study's required sample size is 396 patients at 90% power (stratified with equal sufficiency across a 5 × 5 alcohol assessment table by severity category) for statistical significance of *p* < 0.05, we targeted a sample of 20–30 patients for the pilot study's purpose.

### Data collection

Two fieldwork nurses obtained consent for five alcohol assessment tests: 1) a *venous blood sample*, using enzyme immunoassay to test for BAC, used to validate the presence of alcohol and considered as the gold standard for this study, instead of GC,^5,12^ which was more costly. The venous blood samples were sent via courier to a laboratory for testing; 2) a *clinical observational* assessment (motor coordination, speech impairment, horizontal gaze nystagmus, etc.) for mild/moderate/severe or no alcohol intoxication through the use of a Likert scale and ICD-10 Y91 codes^6^; 3) *active Evidentiary Breath Alcohol Testing (EBAT)* using a validated, South African National Accreditation System and South African Bureau of Standards (SABS) approved instrument,^13–15^ of which the minimum level detected was calibrated at 0.03 mg/l BrAC when blowing through a mouthpiece; 4) SABS approved *passive EBAT* – with no mouthpiece attached, but speaking/breathing near the same device used for active testing, with alcohol indicated as ‘yes/no’; and 5) *a finger prick test* for capillary/whole blood, with a blood collector inserted to a rapid alcohol meter for a digital reading, utilizing fuel cell technology. The injury intent (violence, road traffic, etc.), mechanism (firearm, pedestrian, etc.), age, sex, referral hospital, time of alcohol assessment tests and patient study identification (ID) number were also captured on a Tablet, using a Kobotools platform.^16^ The study questionnaire was adapted from the WHO Collaborative Study on Injuries and Alcohol,^4^ used in a global multi-country study. To test courier logistics, the time of blood withdrawal was captured within the survey questionnaire. In addition, the time of blood sample collection was recorded in a courier log, and the centrifugation time was reported within the laboratory. Blood alcohol concentration test results were merged with these variables by patient study ID using Stata version 17.^17^ Once the pilot study's data collection was complete, the fieldwork nurses provided feedback on logistics regarding use of the alcohol assessment tools. This included feedback on ease of use when conducting the alcohol assessment tests and the need to clean the devices.

### Data analysis

To assess logistics from the pilot study, the time between courier collection after venous blood withdrawal and centrifugation in the laboratory was analysed using Stata version 17^17^ to determine if centrifugation occurred in less than 2 h to preserve the alcohol in the plasma. To validate the alcohol assessment tools, variables analysed included the enzyme immunoassay blood test, active- and passive EBAT, and finger prick test for alcohol. Age, sex and injury intent were also analysed. Ethics approval (EC005–2/2022) was obtained from the South African Medical Research Council's ethics committee. See Petersen Williams et al.^18^ for further detail on the method for the main validation study in progress.

## Results

During the pilot study, we recruited 20 eligible patients, of which 15 were male. The 20 eligible patients were aged between 20 and 44 years, of which 13 were referred from other primary or secondary hospitals. The average time between venous blood withdrawal, courier collection and laboratory centrifugation, was 53 min.

Four patients (20%) tested positive for BAC, of which three cases were road traffic injury-related and one related to violence (Table 1). Positive BAC levels varied between mild and moderate severity, using the enzyme immunoassay blood test. The validated, SABS-approved, passive EBAT ‘yes/no’ results and the active EBAT's digital reading corresponded well with the BAC results from the enzyme immunoassay blood test.

**Table 1.** BAC levels vs. passive and active EBAT readings.

**Injury intent for positive BAC cases**	Blood alcohol concentration **(BAC), g/100 ml (*N* = 20)**	**Passive EBAT indicator (*N* = 18)**		**Active breath alcohol (BrAC) level, mg/**l**, or reason for no result**
		**Yes**	**No**	
**Road traffic**	0.03 (*n* = 1)	1	-	No cooperation
**Road traffic**	0.04 (*n* = 1)	1	-	0.00^a^
**Road traffic**	0.14 (*n* = 1)	1	-	0.40^b^
**Violence**	0.17 (*n* = 1)	1	-	Patient unresponsive
	Zero blood alcohol detected (*n* = 16)	1	13	Not available^c^
	Total (*N* = 20)	**5**	**13**	

BAC: blood alcohol concentration; BrAC: breath alcohol concentration; EBAT: Evidentiary Breath Alcohol Testing.

^a^Active BrAC reading of 0.00 mg/l (not detected) and BAC of 0.04 g/100 ml is within the legal driving limit of 0.05 g/100 ml.

^b^Active BrAC reading of 0.40 mg/l and BAC of 0.14 g/100 ml corresponds to the validated EBAT conversion chart BAC level of 0.084–0.088 g/100 ml.

^c^Zero blood alcohol but no active EBAT results available: reasons were noted as ‘due to injury severity’ for only two cases. No reason stated otherwise.

**Figure 1.**
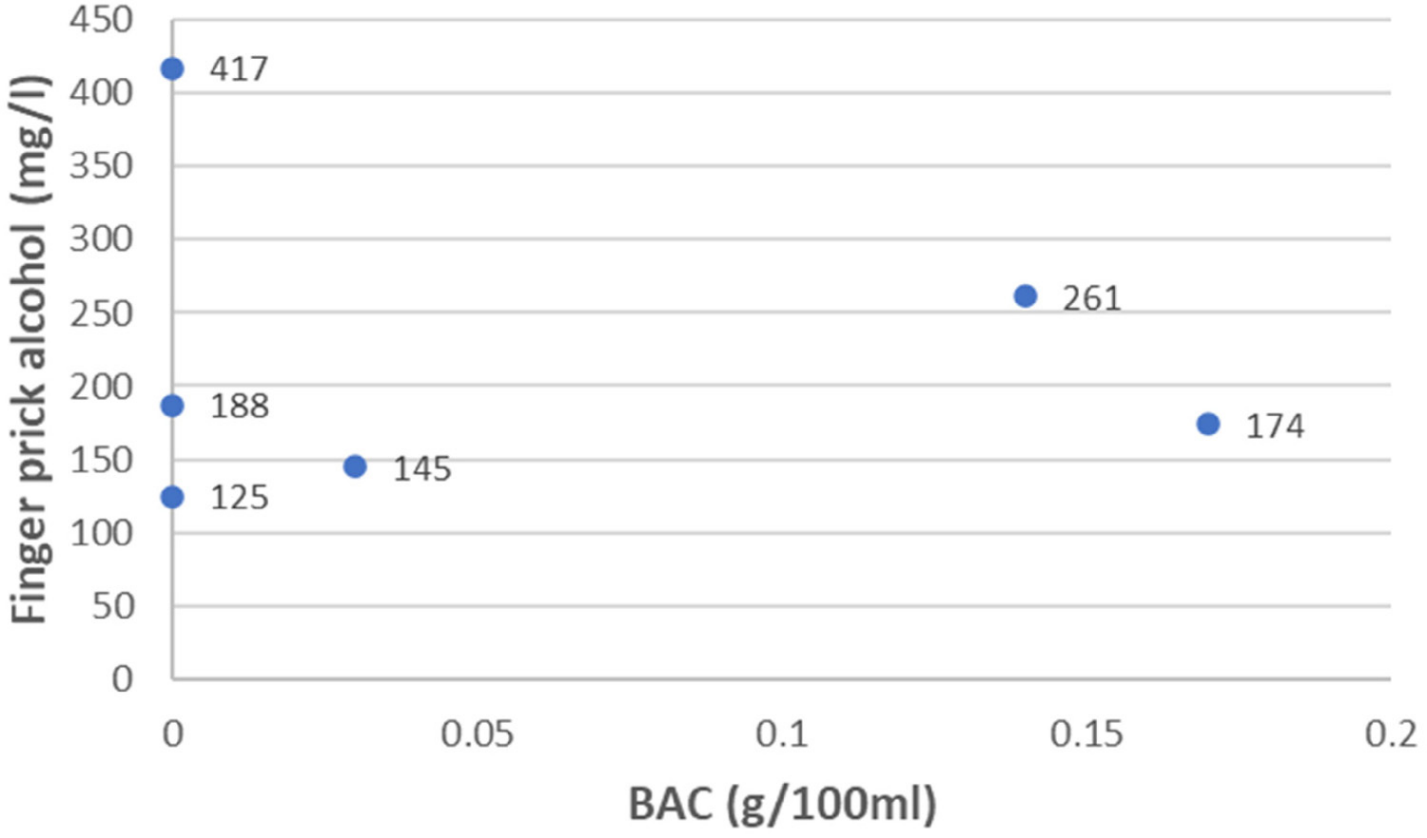
Blood alcohol concentration (BAC) (g/100 ml) vs Finger prick alcohol test^d^ result.

^d^Finger prick conversion chart indicates 100 mg/l = 0.01 g/100 ml BAC and 500 mg/l = 0.05 g/100 ml BAC (SA legal driving limit for alcohol).

None of the finger prick alcohol test results (measured in mg/l) corresponded with those of the enzyme immunoassay blood test (Figure 1). Fieldwork nurses in the ER also found the finger prick procedural method and cleaning of the machine for alcohol analysis before each use cumbersome and time-consuming.

## Discussion and conclusion

The pilot study was highly beneficial as an early indicator of practical and logistic barriers ahead of the more extensive, main validation study in progress. The finger prick procedural method was deemed unsuitable for rapid use in ER settings with high injury caseloads. The discrepancies in finger prick test results, compared to the enzyme immunoassay blood test, were the main reasons for excluding this alcohol assessment tool from the main study. Had we not identified this, we would have incurred further costs to procure more stock. In addition, the finger prick method was not deemed optimal for use, as hand sanitizer has been more frequently used since the outbreak of COVID-19 and could have influenced the results. Conversely, the passive EBAT results show early promise of a cost-effective and easy-to-use alcohol assessment tool for routine testing. This could be most feasible for use in high-volume injury-related ER settings, as in SA, instead of expensive gold-standard blood tests. We would, however, have to verify this with a larger sample size during the current study. The pilot study also identified the need for further testing of the clinical observational assessment using ICD-10 Y91 codes, compared to the enzyme immunoassay blood test, during the main study with a larger sample size.

In addition, we were able to identify that logistically and practically, testing for alcohol in injured patients is better suited in primary and secondary health care settings, where patients are more likely to first present for treatment to avoid exclusion of cases due to time-lapsed delays. The location for the current validation study was hence amended to a secondary, district-level hospital in Cape Town, where patients are treated before tertiary hospital referral, if needed. The current validation study will inform which method of alcohol assessment will be used in the next study phase^18^ for field testing the utility of such instruments in everyday routine health practice in ERs. The suitability for routine use on injured patients will be discussed with expert stakeholders and will conclude with recommended alcohol assessment tools for national scale-up in ER settings.
